# Effects of treatment with *Astragalus Membranaceus* on function of rat leydig cells

**DOI:** 10.1186/s12906-015-0776-3

**Published:** 2015-08-01

**Authors:** Xiaolong Jiang, Xia Cao, Yang Huang, Jianwei Chen, Xiaolei Yao, Miaomiao Zhao, Yan Liu, Jinzhu Meng, Pengfei Li, Zhiyan Li, Jianbo Yao, George W Smith, Lihua Lv

**Affiliations:** College of Animal Science and Technology, Shanxi Agricultural University, No.1 Mingxian Nan Road, Taigu, 030801 China; College of Life Science, Shanxi Agricultural University, Taigu, Shanxi 030801 China; Pharmaceutical Analysis Laboratory of Fudan University, Shanghai, 200000 China; Laboratory of Mammalian Reproductive Biology and Genomics, Michigan State University, East Lansing, MI 48824 USA; Departments of Animal Science, Michigan State University, East Lansing, MI 48824 USA; Departments of Animal Physiology, Michigan State University, East Lansing, MI 48824 USA; Division of Animal and Nutritional Sciences, West Virginia University, Morgantown, WV 26506 USA

**Keywords:** *Astragalus membranaceus*, Leydig cell, SOD and GPx, *Bax*, *Bcl-2*, Testosterone

## Abstract

**Background:**

*Astragalus membranaceus* (*AM*) is a Chinese traditional herb which has been reported to have broad positive effects on many diseases, including hepatitis, heart disease, diabetes and skin disease. *AM* can promote cell proliferation, increase the activities of superoxide dismutase (SOD) and glutathione peroxidase (GPx), and inhibit apoptosis by regulating the transcription of proto-oncogenes controlling cell death. While *AM* is included in some commercially available “testosterone boosting supplements”, studies directly testing ability of *AM* to modulate testosterone production are lacking. In the present study, we examined the effects of *AM* on Leydig cell function *in vitro*.

**Methods:**

Rat Leydig cells were purified and treated with *AM* at different concentrations (0 μg/mL, 10 μg/mL, 20 μg/mL, 50 μg/mL, 100 μg/mL and 150 μg/mL) and cell counting-8 (CCK-8) assay, Enzyme-linked immunosorbent assay, quantitative real time PCR and analysis of activities of SOD and GPx were done respectively.

**Results:**

Treatment with 100 μg/mL (*P* < 0.05) and 150 μg/mL *AM* (*P* < 0.01) significantly increased Leydig cell numbers. Treatment with *AM* (20 μg/mL, 50 μg/mL and 100 μg/mL) significantly increased testosterone production (*P* < 0.01). In addition, increased Leydig cell SOD and GPx activities were observed in response to 20 μg/mL and 50 μg/mL *AM* treatment (*P* < 0.01). Furthermore, expression of *Bax* mRNA was significantly decreased (*P* < 0.01), and the ratio of *Bcl-2/Bax* mRNA was significantly increased in response to 20 μg/mL *AM* in the culture medium (*P* < 0.05).

**Conclusions:**

Results supported a beneficial effect of *AM* on multiple aspects of rat Leydig cell function *in vitro* including testosterone production.

## Background

*Astragalus membranaceus* (*AM*), a well-known Chinese medicinal plant, has been widely used as a traditional prescription medicine for treatment of various diseases, including hepatitis [1], diabetic nephropathy [2, 3], cardiovascular disorders [4, 5] and skin diseases [6]. *AM* has various favorable pharmacological effects including anti-fibrotic [1, 7–9], anti-oxidant [10], anti-apoptotic [11], anti-inflammatory [12] and immune-enhancing properties [13]. *AM* inhibits apoptosis mainly by regulating the expression of *Bax* and *Bcl-2,* members of the B cell leukemia protein (Bcl)-2 family that controls the intrinsic apoptotic pathway [14]. A previous study has shown that glucose-induced podocyte apoptosis was inhibited by intravenous *AM* injection *in vivo* and *AM* treatment *in vitro* with effects linked to down regulation of *Bax* expression and up-regulation of *Bcl-2* expression [15]. Collectively, these observations indicate that *AM* has therapeutic effects in the context of various types of disease.

Although numerous studies support potential medicinal value of *AM*, its effects on the reproductive system have not been well investigated. Limited studies have demonstrated effects of *AM* on the motility of human spermatozoa *in vitro* [16] and amelioration of reproductive toxicity induced by cyclophosphamide in male mice [17] and sperm abnormalities in cadmium-treated rats [18]. Hong *et al.* tested effects of 18 types of Chinese herbs on semen parameters and only *AM* aqueous extract showed a significant stimulatory effect on the motility of human spermatozoa [16].

Leydig cells are distributed in the loose connective tissue between the seminiferous tubules, accounting for 2 %-4 % of testicular cells. Their main physiological function is to produce testosterone. Adult Leydig cells secrete testosterone required for the onset and maintenance of spermatogenesis [19]. However, studies directly testing effects of *AM* on Leydig cells and ability of *AM* to modulate testosterone production are lacking.

In the present study, we examined the effects of *AM* injection on rat Leydig cells, which supported a stimulatory effect on multiple aspects of rat Leydig cell function *in vitro*.

## Methods

*Materials* All materials were obtained from Huaxia Biotech (Beijing, China) unless stated otherwise.

*Animals* Healthy male rats (n = 3) with average weight of 280-300 grams at 50-60 days of age were obtained from the Central Animal Services, Fudan University. Animals were euthanized by CO_2_ inhalation followed by cervical dislocation. All animal procedures were performed with approval of the Fudan University institutional animal care and use committee and in conformity with the guidelines for the care and use of laboratory animals formulated by the Ministry and Science and Technology of China (The Ministry and Science and Technology of the People’s Republic of China, Beijing, China, 2006).

*AM solution preparationAM* injection was purchased from Wu Jing Hospital (Shanghai, China), and each was loaded 100 mL which was equivalent to 20 grams of raw herb. *AM* injection was diluted in dimethyl sulfoxide (DMSO) (Sigma, Shanghai, China) at 10 μg/mL, 20 μg/mL, 50 μg/mL, 100 μg/mL and 150 μg/mL respectively for cell culture.

*Isolation and purification of rat Leydig cells* Testes were excised sterilely from male rats (n = 3) following euthanasia and placed in PBS (pH 7.0-7.2). After washing 2 times with PBS, the testes were placed in DMEM. After removing the epididymis, fat and tunica albuginea, the testes were shredded into small pieces and transferred to a 50 mL centrifuge tube containing DMEM. Leydig cells were dispersed by pipetting followed by centrifugation at 800 rpm for 10 min at 4 °C. Pellet was resuspended in medium followed by centrifugation twice at 600 rpm and 4 °C for 2 min. Supernatant was collected and the cells were seeded into culture dishes for 12 h at 37 °C with 5 % CO_2._ Culture medium was then replaced and cells cultured for another 12 h with Leydig cell purity assessed as described below.

*3β-hydroxysteroid dehydrogenase (3β-HSD) staining* The purity of cells was determined by 3β-HSD staining. After above described 24 h culture, media was removed and cells were removed from culture dish and placed in suspension. The Leydig cell suspension was incubated for 1 h at 37 °C with 1 mg/mL NBT (nitroblue tetrazolium), 3 mg/mL NAD^+^ (Nicotinamide Adenine Dinucleotide), 2 mg/mL DHEA (dehydroepiandrosterone) and1.6 mg/mL nicotinamide in 0.1 M PBS (Phosphate Buffered Saline). Stained cells were washed with PBS once and fixed in 10 % formaldehyde - 50 % ethanol (v/v) for 30 min, after which the cells were sedimented and washed two times. A drop of resuspended cell suspension was placed on a glass microscope slide. After drying, the percent of positive cells with a distinct blue reaction product were observed under a microscope (Olympus, Hong Kong, China).

*Culture of Leydig cells and AM treatment* Purified Leydig cells were seeded in either 96-well culture plates (3 × 10^3^ cells/well) or 24-well culture plates (2 × 10^4^ cells/well) and cultured in 90 % DMEM plus 10 % FBS and 1 % Penicillin-Streptomycin solution at 37 °C and 5 % CO_2_. Treatments consisted of culture medium without (control group with equivalent DMSO instead of *AM* injection) or with different concentrations of *AM* injection (10 μg/mL, 20 μg/mL, 50 μg/mL, 100 μg/mL, 150 μg/mL). 48 h after treatment, the cells and/or culture medium were collected and used in various assays described below. All experiments were repeated 3 times using testes obtained from different rats on different days.

*Cell proliferation assay* Cell proliferation was detected using a CCK-8 (cell counting kit-8) assay kit according to the manufacturer’s instructions (Dojindo, Shanghai, China). After above described 48 h culture, the culture medium was collected and Leydig cells were washed two times with 0.1 M PBS. Then 10 μL of CCK-8 reagent was added to each well and incubated at 37 °C for 2 h. The WST–8 (2 - (2 - methoxy - 4 - (phenyl) - 3 - (4 - (phenyl) - 5 - (2, 4 - sulpho benzene) - 2 H - tetrazolium monosodium salt) in the reagent can be reduced to orange-yellow formazan by dehydrogenase, which is proportional to the number of viable cells. Absorbance at 450 nm was recorded using a microplate reader (Thermo Scientific, Shanghai, China). A standard curve was designed using Leydig cell suspension with different dilution rates to calculate the viable cell numbers in each sample.

*Testosterone Enzyme-Linked Immunosorbent assay (ELISA)* Concentrations of testosterone in culture medium were detected using a Rat Free testosterone ELISA kit following the manufacturer’s instructions (Zhongtianjingwei Science and Technology Co., LTD, Beijing, China). The ELISA plates were read with a microplate reader (Thermo Scientific, Shanghai, China) to record the optical densities and testosterone concentrations derived from standard curve.

*Assays for SOD and GPx activities* After removing the medium, Leydig cells were suspended in 10 mM PBS (pH 7.0-7.2, 100-200 μL/10^6^ cells) and cells lysed via homogenization. The mixture was centrifuged at 4,000 rpm for 10 min at 4 °C and supernatants (20-100 μg protein per sample according to the reagent specification) used for measurement of activity of SOD and GPx using the Total Superoxide Dismutase Assay kit (Jiancheng Bioengineering Institute, Nanjing, China) with WST-8 and Cellular Glutathione Peroxidase Assay Kit respectively (Jiancheng Bioengineering Institute, Nanjing, China). The activity of SOD and GPx was adjusted according to the protein concentrations in different samples.

*Quantitative real time PCR* Total RNA was isolated from the Leydig cell lysates using the miRNeasy mini kit following the manufacturer’s protocol (Qiagen, Shanghai, China). The integrity and concentration of total RNA were measured by agarose gel electrophoresis and Nanodrop-1000 spectrophotometer (Gene Company Limited, Hongkong, China), respectively. Total RNA (500 ng/sample) was then converted to cDNA using the iScript cDNA synthesis kit following the manufacturer’s instructions (Thermo Scientific, Shanghai, China). cDNA was diluted using nuclease free water to a final volume of 40 μL.

Quantitative real time PCR (qRT-PCR) was performed using a 20 μL reaction volume containing 10 μL of SYBR® *premix Ex Taq*™ II(Ruian Biotech., Shanghai, China), 0.4 of ROX Reference Dye II, 0.8 μL each of forward and reverse primer, 2 μL of cDNA and 6 μL of nuclease free water. Reactions were run on a 7500 Real Time PCR system (Thermo Scientific, Beijing, China) for 45 cycles of 95 °C for 15s followed by 60 °C for 1 min. *GAPDH* gene was used as the endogenous control. Primers were designed using Primer 3 (http://primer3.ut.ee/). The primers were as follows: *GAPDH*, 5′- TGGGTGTGAACCACGAGA -3′ (forward) and 5′-GGCATGGACTGTGGTCATGA -3 (reverse); *Bax*, 5′- AGGATGCGTCCACCAAGAAGC -3′ (forward) and 5′-CGGAAGAAGACCTCTCGGGG-3′ (reverse); *Bcl-2*, 5′- GGAGCGTCAACAGGGAGATG-3′ (forward) and 5′- CAGCCAGGAGAAATCAAACAGA -3′ (reverse). The relative mRNA expression level of *Bax* and *Bcl-2* was calculated using the comparative 2^−ΔΔCT^ method [20]. Here, we chose 20 μg/mL *AM* as the treatment, because the results of preliminary test indicated that *AM* in concentration of 20 μg/mL can significantly improve the biological function of Leydig cells, and the concentration is more economical in a real application.

*Statistical analysis* All data were analyzed in one way ANOVA using SPSS computer software (IBM, USA). Effects of *AM* treatment (control versus 20 μg/mL *AM*) on *Bcl-2* and *Bax* mRNA were analyzed by Student’s *t*-test. Means were separated using Tukey’s test. Data are presented as mean ± SE.

## Results

**Fig. 1:**
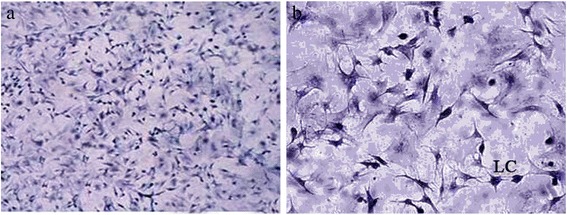
3β-HSD staining of Leydig cells. **a** Magnification, 100×; **b** Magnification, 400×. LC, Leydig cell

*Leydig cell purity* To assess the purity of cultured Leydig cells, 3β-HSD staining was performed. The results showed that the purity of cultured Leydig cells was > 95 % (Fig. 1).

**Fig. 2:**
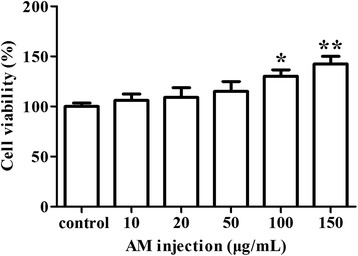
Effect of *AM* treatment on viable Leydig cell numbers. Cells were treated with 0 μg/mL, 10 μg/mL, 20 μg/mL, 50 μg/mL, 100 μg/mL and 150 μg/mL of *AM*. Cell viability was analyzed by CCK-8 assay. Results are depicted as mean +/- SE * *P* < 0.05, ** *P* < 0.01

*AM treatment increased Leydig cell numbers* To investigate the effect of *AM* injection on Leydig cells, numbers of viable cells were determined by CCK-8 assay 48 h after *AM* treatment. Compared with the untreated control, numbers of viable cells increased within a certain range of concentration, particularly at the concentrations of 100 μg/mL (*P* < 0.05) and 150 μg/mL (*P* < 0.01) (Fig. 2). The result suggested that addition of *AM* increases numbers of viable Leydig cells 48 h after treatment.

**Fig. 3:**
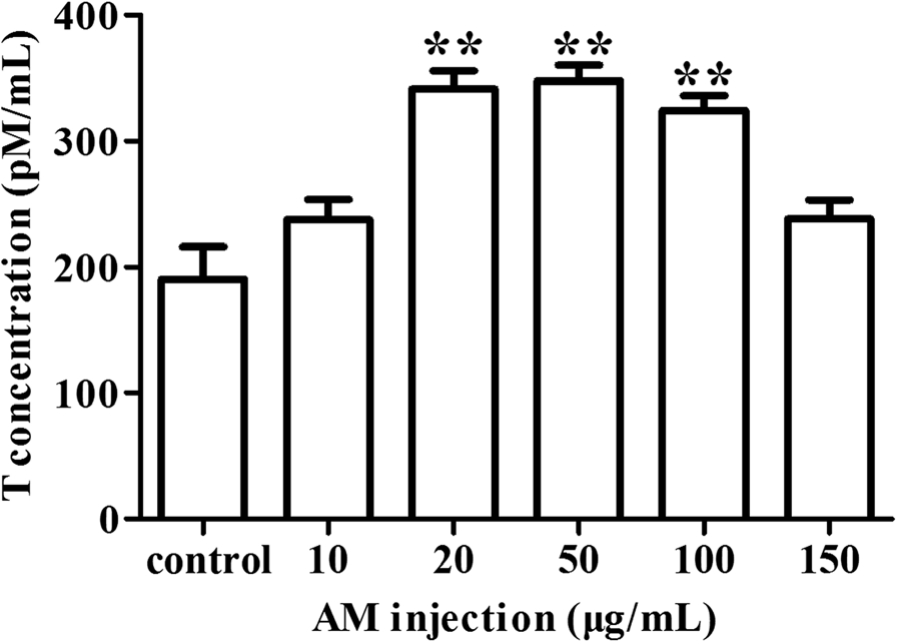
Effect of *AM* treatment on testosterone production in Leydig cells. Cells were treated with 0 μg/mL, 10 μg/mL, 20 μg/mL, 50 μg/mL, 100 μg/mL and 150 μg/mL of *AM*. Testosterone production in culture medium was determined by ELISA (enzyme-linked immuno sorbent assay). Results are depicted as mean +/- SE, * *P* < 0.05, ** *P* < 0.01

*AM treatment increased Leydig cell testosterone production* To determine potential effects of *AM* on the production of testosterone in Leydig cells, cells were treated with increasing concentrations of *AM*. Significantly higher concentrations (*P* < 0.01) of testosterone were observed in culture media of cells treated with 20 μg/mL, 50 μg/mL, and 100 μg/mL of *AM* injection for 48 h (compared with untreated control; Fig. 3), suggesting certain stimulatory effects of *AM* injection on Leydig cell testosterone production. Intra- assay and inter- assay coefficients of variation were < 11% and < 9 %, respectively.

**Fig. 4:**
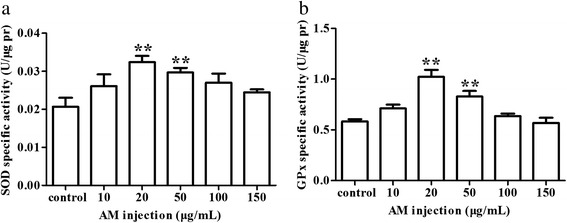
Effect of *AM* treatment on the activities of SOD and GPx in Leydig cells. Cells were treated with 0 μg/mL, 10 μg/mL, 20 μg/mL, 50 μg/mL, 100 μg/mL and 150 μg/mL of *AM*. **a** SOD activity. **b** GPx activity. Results are depicted as mean +/- SE, * *P* < 0.05, ** *P* < 0.01

*AM injection treatment increased SOD and GPx activities* To determine the effect of *AM* on the antioxidant defense system, the activities of SOD and GPx were detected in cultured Leydig cells 48 h after *AM* treatment. As shown in Fig. 4, the activities of SOD and GPx were significantly increased in Leydig cell cultures treated with 20 μg/mL and 50 μg/mL of *AM* relative to untreated controls (*P* < 0.01). Results indicated *AM* treatment could increase Leydig cell antioxidant activity within a certain range of concentration.

**Fig. 5:**
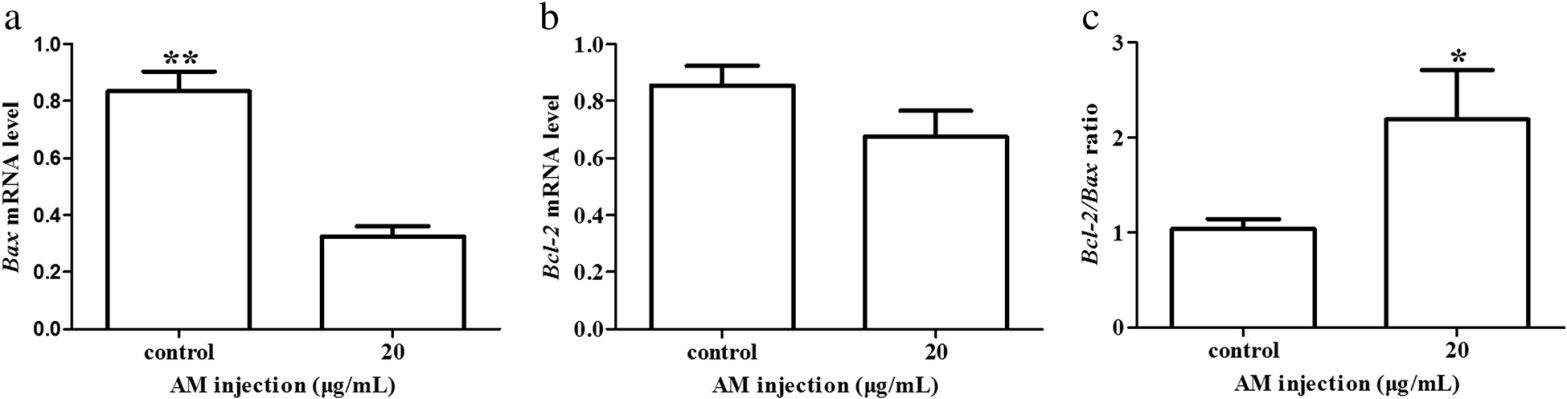
Effect of *AM* treatment on the expression of *Bax* and *Bcl-2* in Leydig cells. qRT-PCR was performed to determine the abundances of *Bax* (**a**) and *Bcl-2* (**b**) mRNA in Leydig cells treated with 20 μg/mL of *AM*, and to further determine the effect of *AM* treatment on expression level of *Bax* and *Bcl-2*, we calculated the ratio of *Bcl-2* to *Bax* (**c**). Expression of *Bax* and *Bcl-2* mRNA was normalized relative to the abundance of *GAPDH* mRNA. Results are depicted as mean +/- SE (n = 3). * *P* < 0.05, ** *P* < 0.01

*Effect of AM on Bax and Bcl-2 mRNA expression in AM treated Leydig cells* To further elucidate the potential effects of *AM* on Leydig cell numbers, the expression of apoptosis-related genes *Bax* and *Bcl-2* in Leydig cells treated with 0 μg/mL or 20 μg/mL of *AM* injection for 48 h were analyzed using qRT-PCR. Results showed in Fig. 5 indicated the expression of *Bax* mRNA was significantly reduced in *AM* treated group (*P* < 0.01) compared with the untreated control, while the expression of *Bcl-2* mRNA had no obvious change between *AM* treated group and the untreated control (*P* > 0.05). However, the ratio of *Bcl-2/Bax* mRNA was significantly higher in the *AM* treated group versus the untreated control (*P* < 0.05).

## Discussion

Many studies have suggested a positive role for *AM* in the treatment of various diseases. In the present study, we investigated the effect of *AM* injection on function of rat Leydig cells. According to the instructions for *AM* injection and relevant reference [21], we at first used *AM* concentrations that ranged from 0-80 μg/mL to take the preliminary test. The result showed that there was no significant difference in cell number among these concentrations (the result was not provided). After several times of preliminary test and adjusting the concentration gradient, finally we chose 0 μg/mL, 10 μg/mL, 20 μg/mL, 50 μg/mL, 100 μg/mL, 150 μg/mL as the test concentration gradient.

Several studies have reported stimulatory effects of Astragaloside (main compound extracted from *AM*) [4, 22] on cell proliferation, which was also observed in this study. Leydig cells influence sexual maturation of males by secreting testosterone, which is influenced by cell number and rate of steroidogenesis. However, significant changes in cell proliferation and testosterone production did not occur at the same dosage of *AM*. The increase in testosterone production occurred at lower concentrations of *AM* than those demonstrated to increase Leydig cell numbers. Results suggested direct effects of *AM* on regulation of steroidogenesis independent of modulation of cell numbers.

Apoptosis is regulated by various factors, among which pro- and anti-apoptotic proteins of Bcl-2 family play an important role. Relative to the absolute expression of either gene alone, the ratio of *Bcl-2* to *Bax* is regarded as a better determinant to measure cell survival [23]. In the present study, we found significantly decreased *Bax* expression in the *AM* treated Leydig cells, which is consistent with a previous study demonstrating similar effects of *AM* on *Bax* expression in skin [11]. However, *AM* treatment did not increase Leydig cell *Bcl-2* expression in the current studies. Therefore, the increased ratio of *Bcl-2/Bax* in *AM* treated group may be attributed to the inhibitory effect of *AM* on *Bax* mRNA. A previous study demonstrated that intravenous injection of Astragaloside down-regulated *Bax* mRNA to reduce apoptosis in a rodent acute kidney injury model [3].

SOD catalyzes the dismutation of the superoxide anion, and GPx mediates the breakdown of hydrogen peroxide. These enzymes, as well as other antioxidant enzymes, are the main components of antioxidant defense system that possesses high potency to scavenge reactive oxygen species free radicals that are detrimental to cell survival by affecting cellular signaling pathways and gene expression. In the present study, we detected increased activities of SOD and GPx in the *AM* treated Leydig cells, supporting the antioxidant role of *AM*. Previous studies linked the therapeutic effects of *AM* on myocardial ischemia [24], ischemic brain injury [25], hemorrhagic shock-reperfusion injury of intestinal mucosa [26] and the epithelial-to-mesenchymal transition in diabetic kidney disease [21] to increased activities of antioxidant enzymes such as GPx and SOD. A large body of evidence indicates antioxidants can suppress apoptosis. Hence, it is plausible that the increase in Leydig cell numbers is linked, at least in part, to stimulatory effects of *AM* on SOD and GPx activity resulting in reduced apoptotic cell death.

Cell proliferation is a process regulated by lots of factors. The SOD and GPx activity was just one aspect that could be considered to evaluate the growth of Leydig cells. Perhaps *AM* at the concentrations of 20 μg/mL and 50 μg/mL was optimal for the SOD and GPx activity of Leydig cells although it had no obvious promotion for cell proliferation. With the increase of concentration, the amount of *AM* was too much for the SOD and GPx, so the activity decreased, but the number of Leydig cells was significantly increased in the process. Perhaps all above lead to the result that the treated group in which there was a significant difference of Leydig cell number was not consistent with that of SOD and GPx activity. Results suggested direct effects of *AM* on regulation of SOD and GPx activity independent of modulation of cell numbers.

Although the study preliminarily indicated that *AM* injection has a stimulatory effect on multiple aspects of rat Leydig cell function *in vitro*, it had some limitations, such as the design of *AM* injection gradient, and this was just the results of cell culture level. Therefore, lots of work and study should be done to elucidate the exact mechanism of *AM* promoting the function of Leydig cells.

## Conclusions

In the present study, we investigated the influence of *AM* injection on cell numbers and testosterone production of rat Leydig cells cultured *in vitro.* The results suggested that *AM* injection has a favorable effect on the function of Leydig cells. The study provides a foundation for future studies to understand the mechanisms responsible for stimulatory effects of *AM* injection on Leydig cells and whether *AM* treatment *in vivo* can enhance indices of male fertility.
